# Spatial and temporal variability of carbon dioxide fluxes in the Alpine Critical Zone: The case of the Nivolet Plain, Gran Paradiso National Park, Italy

**DOI:** 10.1371/journal.pone.0286268

**Published:** 2023-05-30

**Authors:** Sara Lenzi, Marta Magnani, Ilaria Baneschi, Mariasilvia Giamberini, Brunella Raco, Gianna Vivaldo, Antonello Provenzale

**Affiliations:** 1 Institute of Geosciences and Earth Resources, CNR, Pisa, Italy; 2 INFN, Torino, Italy; Tennessee State University, UNITED STATES

## Abstract

The dynamics of carbon dioxide fluxes in the high-altitude Alpine Critical Zone is only partially understood. The complex geomorphology induces significant spatial heterogeneity, and a strong interannual variability is present in the often-extreme climatic and environmental conditions of Alpine ecosystems. To explore the relative importance of the spatial and temporal variability of CO_2_ fluxes, we analysed a set of in-situ measurements obtained during the summers from 2018 to 2021 in four sampling plots, characterised by soils with different underlying bedrock within the same watershed in the Nivolet plain, in the Gran Paradiso National Park, western Italian Alps. Multi-regression models of CO_2_ emission and uptake were built using measured meteo-climatic and environmental variables considering either individual years (aggregating over plots) or individual plots (aggregating over years). We observed a significant variability of the model parameters across the different years, while such variability was much smaller across different plots. Significant changes between the different years mainly concerned the temperature dependence of respiration (CO_2_ emission) and the light dependence of photosynthesis (CO_2_ uptake). These results suggest that spatial upscaling can be obtained from site measurements, but long-term flux monitoring is required to properly capture the temporal variability at interannual scales.

## 1. Introduction

Each year, terrestrial ecosystems globally contribute to the net fixation of about 3.1 Gt of carbon from the atmosphere, corresponding to about one third of the anthropogenic CO_2_ emissions (from both fossil fuel combustion, 9.6 GtC, and land-use change, 1.2 GtC) [1]. Under the effects of climate change, the ongoing temperature rise may induce a longer growing season, thus increasing productivity and CO_2_ uptake, but at the same time it may increase evaporation and plant stomatal closure, thus reducing productivity. Similar contrasting effects on plant activity may be caused by the accumulation of CO_2_ in the atmosphere [2]. Emissions from soil microbial respiration may also either increase owing to higher temperatures, and possibly to the atmospheric CO_2_ enrichment, or decrease in response to droughts and to the depletion of soil organic carbon (SOC) [3, 4]. The net balance between these processes is expected to be biome-specific for both CO_2_ uptake and emission [3, 5].

In high-mountain environments, owing to the elevation dependence of the temperature rise [6, 7] and the often extreme local conditions [8], the impacts of climate change can be especially severe, leading to variations in the phenology and distribution of vegetation [9], changes in carbon and water fluxes [10], and local extinction of endemic and/or endangered species [11]. For this reason, and for the wide extent of mountain ecosystems all over the world [12], it is especially important to understand what are the drivers of CO_2_ fluxes in high-elevation habitats, what is the interannual variability of carbon fluxes generated by interannual changes in meteo-climatic conditions, and whether modelling descriptions adopted for specific areas can be upscaled to other locations and larger areas.

Terrestrial ecosystems respond to climate change also through modifications in the Critical Zone (CZ), the Earth’s surface layer including soil, vegetation and low atmosphere [13, 14]. The CZ is the ‘biogeochemical reactor’ of our planet [15, 16]; in particular, it is the main player in CO_2_ exchanges between the land and the atmosphere. Therein, the flux dynamics can be studied using field measurements, based on either direct techniques, such as Eddy Covariance methods and accumulation chambers, or remote sensing products, such as satellite observations estimating primary productivity. The measurements provided by the different approaches are often complementary, and each of them provides a specific piece of information on the complex dynamics of the Critical Zone, thus contributing to the upscaling problem at different levels. For example, several satellite methods have been developed to monitor the phenological patterns in mountain grasslands, despite these methods being limited by a long satellite revisit time, which affects the capabilities of the algorithms to reconstruct the time series [17]. On the other hand, direct measurements as Eddy Covariance and automated accumulation chambers could provide high frequency time series, although with limited spatial coverage.

In this work, we explore the spatial and temporal variations of CO_2_ fluxes at landscape scale, focusing on the high-altitude Alpine grasslands of the Nivolet Plain in the Gran Paradiso National Park, western Italian Alps. By means of a portable accumulation chamber, we performed in situ measurements of CO_2_ fluxes over four years and in four sampling plots, characterised by soils with different underlying bedrock and topographic position, within the same watershed. In particular, we expected that 1) the flux behaviour significantly differed between the four plots, for example owing to different soil characteristics, and 2) the interannual variability of CO_2_ flux dynamics could not be fully explained by the interannual variability of the drivers, because the functional dependence of the fluxes on the drivers could vary from year to year. To study the dependence of CO_2_ emission and uptake on meteorological, climatic, and environmental variables, we built empirical, data-driven models which were parameterized either for each plot or for each year.

## 2. Materials and methods

### 2.1 Study site

**Fig 1:**
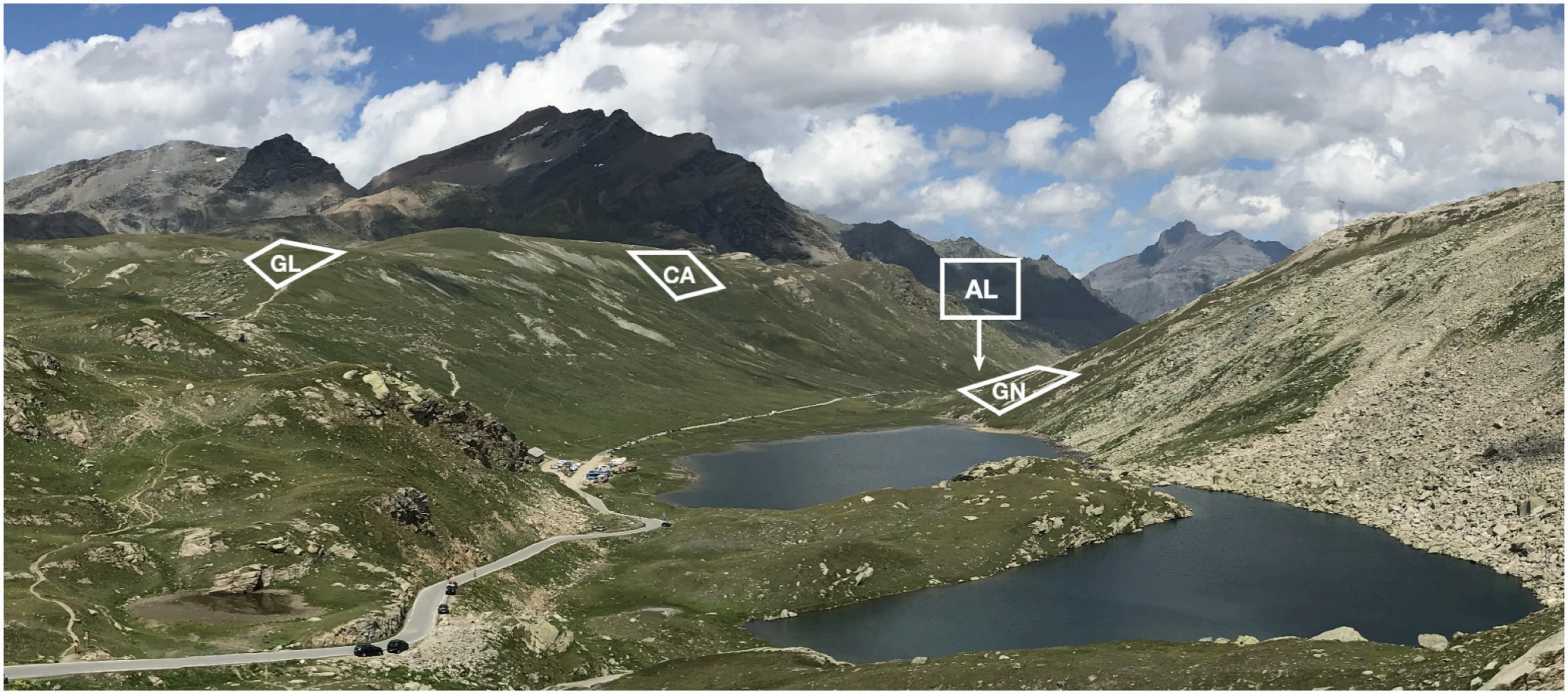
View of the study area in the Nivolet Plain seen from the Nivolet Pass. The four sampling plots are labelled as GL (Glacial), CA (Carbonate), GN (Gneiss) and AL (Alluvial).

The study area is located in the Nivolet Plain (Fig 1) and it is part of the Gran Paradiso National Park (GPNP), in the western Italian Alps. This area is included in the Alpine Critical Zone Observatory (see also https://www.czen.org/content/nivolet-czo) managed by the Italian National Research Council, located at the Nivolet Plain (CZO@Nivolet). The Gran Paradiso National Park is managed by the park authority, which granted access to the study area and authorised the measurement activities for each field campaign.

The Nivolet Plain is a hanging valley whose bottom extends from about 2700 m a.s.l. of the Nivolet Pass to the SW, to about 2300 a.s.l. to NE. During winter (mostly from November to late May) the soil is covered with a thick coat of snow. The highest snow depth recorded at the close weather station located at Serrù lake was 295 cm in the last three years. The mean summer (June-October) temperature ranged between -8 °C and 27 °C (min and max average values in 1962–2021) and the mean annual precipitation was 1174 mm/yr, with an average summer precipitation of 476 mm/yr, in the period 1962–2021. From mid-late June to October, when the snow cover is absent, vegetation undergoes a rapid greening and a subsequent slower transformation towards senescent conditions that usually begins in late August.

Geologically, the Nivolet Plain is located at the tectonic contact between the Gran Paradiso massif and the Piedmont-Ligurian oceanic units (e.g. [18, 19]). The Gran Paradiso massif consists of abundant augen–gneisses (“gneiss occhiadini” Auct.) derived from Permian porphyritic granitoids intruded into metasedimentary rocks (polymetamorphic “gneiss minuti” Auct.). The overlying Piedmont-Ligurian units display remnants of a Mesozoic cover, represented by carbonate rocks (dolostones and marbles) and scarce quartzites. The Nivolet Plain area is drained by the meandering ‘Dora del Nivolet’ stream and clearly recorded Quaternary glacial dynamics. Pleistocene glacial deposits composed of unsorted till and glaciofluvial sediments with interbedded peat layers are mainly preserved on the left flank of the valley.

Leveraging the complex geologic history of the area, we identified four plots within the Nivolet Plain (Fig 1), characterised by different geomorphological characteristics, soil origin and parental material. Each plot measured about 500 m^2^ and, for the purpose of this study, was labelled according to the soil origin and/or underlying bedrock. The first plot, located on the left flank of the valley at an altitude of 2740–2750 m a.s.l. was characterised by soils formed on glacial unsorted tills (called Glacial site, GL, hereafter). On the same hillside at 2750–2760 m a.s.l., we identified the second plot, with soils formed on carbonate rocks (named Carbonate site, CA). On the right flank of the valley, we identified the third plot at 2580–2600 m a.s.l, with soils on gneiss rocks (the Gneiss site, GN). The fourth plot was located on the valley bed, at about 2530–2550 m a.s.l, where the soils were enriched by alluvial and colluvial deposits of the ‘Dora del Nivolet’ river (site Alluvial, AL).

In these four plots, surface soils (up to 10 cm depth) showed relatively different physical and chemical characteristics (S1 Fig). The soils feature a sandy texture, with higher values of sand content in plot GN and AL compared to CA and GL; vice versa, silt content is slightly higher in plot CA and GL compared to GN and AL. Plots GN and GL are respectively enriched in organic carbon and nitrogen compared to the other plots, and these patterns could potentially cause local flux differences [3]. The four plots present similar plant species, whose cover slightly varies across the plots: *Trifolium alpino*, *Ranunculus pyrenaeus* and *Pulsatilla alpina* are abundant in plot AL, *Geum montanum* covers a large area in plots CA and GL, and *Carex curvula* is dominant in plot GN.

### 2.2 Measurement procedure

Flux measurements were based on the accumulation chamber method [20]. The measurement apparatus is composed of a transparent polycarbonate chamber (produced by WEST System SRL, with a height of 31.5 cm and a base area of 363 cm^2^), connected to a LICOR LI-840 IR gas analyser, mounted inside a transportable hard case.

The measurements were performed from 2018 to 2021 during summer months (i.e., from June to October), with 3 to 6 measurement campaigns carried out over each plot and in each year. During each measurement campaign and for each plot, CO_2_ fluxes were measured in M = 20 individual points with a random spatial distribution over the plot, for a total sampling time of 2 hours. Different land cover types were usually contained within the chamber base area, including a mix of small vascular plants (the most common are *Carex spp*., *Trifolium alpinum*, *Pulsatilla alpina* and *Geum montanum*), litter, mosses, likens and bare soil. Each individual measurement is thus the sum of different contributions. Averaging *M* individual point measurements allowed us to estimate the typical flux magnitude in the plots, thus accounting for all the contributions. Such a number of individual measurement points have been shown to prove a stable and statistically significant estimate of the mean flux value over the plot; see [21] for further details.

In each sampling point, a stainless-steel ring was inserted into the ground, to a depth of about 1 cm, and the chamber was leaned on the ring (with a height of 4 cm and a diameter of 21.5 cm). Two consecutive measurements were performed: one with the transparent chamber and one with the chamber covered by a blackout cloth, thus estimating the Net Ecosystem Exchange (*NEE*) and the Ecosystem Respiration (*ER*), respectively. During the measurement, the increase (CO_2_ emission) or decrease (CO_2_ uptake) of CO_2_ concentration inside the chamber was recorded. Excluding the initial transient phases that lasted for about twenty seconds, the concentration vs time series was linearly interpolated over 60 s to estimate the flux. Between the two (*NEE* and *ER*) measurements, the CO_2_ concentration in the chamber was restored to the environmental level. Assuming nearly unchanged environmental conditions between the two consecutive measurements, we calculated the fixation of carbon dioxide due to photosynthesis (i.e., the Gross Primary Production, *GPP*) as *GPP* = *NEE*—*ER*. In this convention, *ER* is positive or null (*ER*≥0), *GPP* assumes null or negative values (*GPP≤*0), and *NEE* can be either positive or negative, depending on whether the flux is dominated by *ER* or *GPP*, respectively.

In addition to the carbon flux measurements, we recorded several basic environmental variables. The soil temperature (*T*_*s*_) and the soil volumetric water content (*VWC*) were measured at a depth of 5 cm outside but in proximity of the collar, while air humidity (*q*) and air temperature (*T*_*a*_), atmospheric pressure (*Pr*) and solar irradiance (*rs*) were measured at a height of 1.5 m above the ground. Soil temperature was measured using a PT100 thermometer, soil moisture using a SM150T delta-t-devices hygrometer. Air temperature and moisture were measured using a LSI Lastem-DMA672.1 termo-hygrometer and solar irradiance by a LSI Lastem-DPA053 pyranometer.

### 2.3 Statistical analysis and model implementation

For the statistical analysis, we first computed the mean and standard deviation of the point flux measurements and environmental variables for each plot and each campaign, thus obtaining an ensemble of N = 91 plot-scale flux and environmental estimates spanning the four years and four plots. This dataset constitutes the full distribution of measurement values to be used in the analysis. To explore the temporal and spatial variability of the fluxes we compared the measurements taken in either different sampling years (aggregating over all sites) or different sampling plots (aggregating over all the years). That is, we used the marginal distributions of the full distribution of plots and years, which allowed us to maintain sufficient statistics in the datasets to be compared.

The subsequent statistical analysis followed three main steps:

1. First, we focused on the mean *ER* and *GPP* flux values and environmental variables. The significance of the difference between the variables recorded in the different plots or the different years was estimated. Since the environmental variables and the fluxes are known to have seasonal and daily patterns in this ecosystem, we also assessed possible differences in the mean day and hour of sampling across different sites and plots. Such a test allowed us to assess whether the differences between years or plots observed in the mean values of the environmental variables and fluxes were induced by the different distribution of measurements across the summer or across the day.
2. Then, we tested the validity of the flux models developed in [21]. Assuming the same general formulation, we explored the possible flux dependencies on the environmental variables (see Sec. 2.3.2).
3. Finally, we studied the significance of differences in model parameters between either plots or years, assessing whether the different subsets belonged to different statistical populations or could be considered as different sub-samplings of the same distribution.

A random shuffling method was used in all the three steps [22]. This method is based on the random shuffling of samples (without repetitions) to generate a surrogate series that is used to perform a double-tailed test on the distribution of the surrogates. In the first step, the shuffling technique was used to assess the significance of the mean differences between plots (or years). We obtained 100 surrogate series by shuffling the data across the two original series, thus mixing values coming from the two different original series. The differences of the mean values were computed for each pair of surrogates, generating a distribution of mean-value differences compatible with the null hypothesis that they are generated purely by statistical fluctuations. Comparing the mean-value difference of the two original series with such distribution using a two-tailed approach, we obtained a P-value corresponding to the probability of obtaining the observed difference purely by chance. The difference is considered statistically significant when P≤0.05. In the second step, the shuffling method was used to test the significance of parameter estimates for each year or site. In this case, we shuffled the samples of the predictors, *x*, and we re-fitted the model to the surrogate series. Thus, we broke the causality nexus between *x* (the independent driver) and *y* (the dependent variable to fit—i.e. the flux) and tested the distribution of the parameters against the null hypothesis that the estimated parameter values were different from 0 purely by chance. Finally, in the third step we estimated the significance of the model parameter differences between pairs of plots or years. This was done by shuffling individual (*x*, *y*) couples across different years or plots, preserving the driver-flux association of each couple. The model was fitted to the two surrogate series, thus obtaining the distribution of parameter difference that allowed us to test the significance of the observed parameter difference. See [21] for further details on the shuffling method. The model performance was evaluated computing the explained variance (${\sigma^{2}}_{expl}$), and the gaussianity of model residuals was tested with the Lilliefors’ test [23]. All the analyses were developed on Matlab R2021b using the intrinsic function for the Lilliefors’ test, while implementing dedicated functions for the shuffling tests. The dataset and the code used in this work are freely available in the Zenodo repository [24].

### 2.4 Carbon flux models

Traditionally, *ER* is modelled with a temperature-dependent exponential [25] and *GPP* with a double hyperbolic, Michaelis-Menten function [26]. These classical drivers were shown to describe the flux variability only loosely at our sampling plots [21], while a similar study in the Arctic tundra showed that they correctly represent the fluxes repeatedly measured over short periods in an individual point [27]. This indicates that other drivers are active at plot scale, owing to the heterogeneity of soil (e.g., moisture) and vegetation characteristics, and a more comprehensive model is thus needed.

The equations of the models proposed in [21] are:

$$ER=\left(a_{0}+a_{1}VWC+a_{2}Pr+a_{3}DOY\right)e^{b_{0}T}+\delta$$ (1)

$$GPP=\frac{F_{0}\alpha_{0}rs}{F_{0}{+\alpha }_{0}rs}\left(A_{0}+A_{1}VWC+A_{2}DOY\right)+\delta$$ (2)

Where *a*_*i*_, *b*_0_, *A*_*i*_, *F*_0_, *α*_0_ are parameters to be fitted and *δ* represents model residuals (i.e., the difference between measured and fitted values). Here, the typical dependence of GPP on Photosynthetic Active Radiation was replaced with a dependence on solar irradiance (rs). As mentioned above, for the purpose of this work we tested Eqs (1) and (2) either on each plot (aggregating over all years) or for each year (aggregating over all sites).

As in the original work [21], we assumed that the parameters of the classical functions (the exponential for *ER* and the Michaelis-Menten for *GPP*) were functions of additional environmental variables, and a generalised model was obtained by Taylor expansions. The additional predictors were selected from the list of the measured environmental variables, complemented by the day of the year (*DOY*, 1–365) and the hour of sampling (*h*, in decimals, obtained as hour+minutes/60). In order to avoid collinearity, environmental variables showing large and significant correlations were not included in the same model. We considered different sets of additional predictors, and we selected the optimal set based on the Akaike Information Criterion (AIC). According to the AIC, the model minimising the AIC should be preferred, because it maximises the model representativeness, as well as its efficiency. By this criterion, we assessed the validity of Eqs (1) and (2) against other possible sets of additional variables.

## 3. Results

### 3.1 Mean values

Table 1 shows the mean values of the flux and environmental variables, for each individual plot (aggregated over years), or each year (aggregated over plots).

**Table 1:** Mean values of environmental variables, day and hour of sampling and flux estimates for each individual year (aggregated over all plots) and for each individual plot (aggregated over all years).

		*T*_*s*_	*T*_*a*_	*VWC*	*rs*	*q*	*Pr*	*h*	*DOY*	*NEE*	*ER*	*GPP*
year	2018	15.9^a^	12.7^ab^	25.8^a^	757.2^a^	52.5^a^	749.1^ab^	13.4^a^	227.6^a^	-2.3^a^	2.9^a^	-5.2^a^
	2019	12.2^b^	11.2^bc^	21.8^ac^	657.8^ab^	62.1^b^	748.6^a^	13.2^a^	229.5^a^	-2.8 ^a^	3.2^a^	-6.0 ^a^
	2020	17.2^a^	14.2^a^	20.1^c^	791.1^a^	55.1^ab^	745.9^a^	13.2^a^	227.4^a^	-5.0^b^	5.1^b^	-10.1^b^
	2021	12.9^b^	9.8^c^	34.1^b^	601.3^b^	62.4^ab^	746.9^b^	14.0^a^	222.3^a^	-4.3^ab^	5.5^b^	-9.7^b^
plot	GL	14.5^AB^	11.1^A^	31.4^A^	724.2^AC^	59.8^A^	741.5^A^	13.7^A^	228.4^A^	-2.9^A^	4.5^A^	-7.3^AB^
	CA	16.6^A^	11.8^A^	23.1^B^	799.0^A^	54.3^A^	740.2^B^	13.3^A^	225.3^A^	-4.0^A^	4.6^A^	-8.6^AB^
	GN	12.9^B^	12.1^A^	27.3^AB^	581.6^B^	62.4^A^	754.1^C^	14.2^A^	225.9^A^	-3.0^A^	3.3^B^	-6.3^A^
	AL	12.6^B^	11.7^A^	22.8^B^	622.2^BC^	57.1^A^	759.1^D^	12.4^A^	225.0^A^	-5.0^A^	4.5^AB^	-9.5^B^
All		14.3	11.6	26.6	687.6	58.6	747.7	13.5	226.3	-3.5	4.2	-7.8

The line ‘All’ refers to the average over all plots (GL = Glacial; CA = Carbonate; GN = Gneiss; AL = Alluvial) and all years. For each variable, significant differences between years are marked by different lowercase letters, while differences between plots are marked by different uppercase letters.

*T*_*s*_: soil temperature (°C); *T*_*a*_: air temperature (°C); *VWC*: soil volumetric water content (%); *rs*: solar irradiance (W m^-2^); *q*: air humidity (%); *Pr*: atmospheric pressure (hPa); *h*: hour of sampling in decimals (hour+minutes/60); *DOY*: day of the year (1–365); *NEE*: net ecosystem exchange (μmol m^-2^ s^-1^); *ER*: ecosystem respiration (μmol m^-2^ s^-1^); *GPP*: gross primary production (μmol m^-2^ s^-1^).

The variables having significant differences between years or plots are grouped in Table 1 using letters (see also S1 and S2 Tables). In general, smaller differences were observed between plots than between years, suggesting that interannual variations are more important than spatial variability across the plots.

Considering each individual variable, no significant differences were observed in the mean *DOY* and hour of sampling neither between years nor between plots, indicating that the measurements were evenly distributed over the sampling season and over the day. This allowed us to compare the average values of environmental variables and carbon fluxes between different years or plots excluding biases caused by a different temporal distribution of the samplings across the summer period or the day.

The warmest year was 2020, followed by 2018 for both air and soil temperature. This corresponded to significant differences in the mean soil temperature between the two groups 2018–2020 and 2019–2021, and similar patterns were observed in the mean annual values of air temperature. No significant differences were obtained in the plot average air temperature and only the soil temperature measured in plot CA differed significantly from the other plots, except for GL; while GL, GN and AL showed statistically similar soil temperatures.

The driest year was 2020 in terms of soil humidity and 2018 in terms of air humidity. The most humid year (2021 for both soil and air humidity) corresponded to the lowest air temperature. Most of the years showed significant differences in the mean annual soil moisture, while air humidity was different between 2018 and 2019, which in turn were both similar to the other years. On the other hand, the mean soil moisture was mostly different between GL and the other plots, except for GN, while the mean air moisture measured in each plot was statistically similar.

The year-to-year patterns of solar irradiance were similar to the air temperature patterns, also in the distribution of significant differences. The mean solar irradiance measured in different plots was statistically similar between pairs of plots, i.e. between GL-CA, GN-AC and GL-AL.

The atmospheric pressure was significantly different between 2021 and the previous years, except 2018; the mean pressure measured in each plot was significantly different from the others, and reflected the altitudinal distribution of the plots.

The *ER* was higher in 2020 and 2021 and both years significantly differed from 2018 and 2019, which were statistically similar between them. The variations of *ER* did not match the observed temperature patterns. In particular, the low air temperature in 2021 was associated with the highest *ER*, and the lowest *ER* in 2018 corresponded to a high mean temperature in the same year. Plots mostly had statistically similar ER, except for GN which was only similar to AL. The ER was maximum in plot CA and minimum in plot GN. Across the plots, emission and temperature did not show similar patterns.

In general, lower (more intense) *GPP* values matched higher (more intense) *ER* values. In analogy with ER, the mean GPP measured in both 2020 and 2021 significantly differed from 2018 and 2019, which were statistically similar between them. The *GPP* patterns did not reflect the patterns in solar irradiance, neither across years nor across plots. The maximum *GPP* was measured in plot AL, followed by plot CA, while the lowest *GPP* was observed in plot GN.

The *NEE* was more intense (i.e. highest in absolute value) in 2020, which showed significant differences with respect to both 2018 and 2019, but was statistically similar to 2021; while no significant differences were observed between plots.

### 3.2 Multivariate models

As a first step, the correlation between environmental variables was assessed. Considering all plots and years (i.e., the correlation between variables for the whole dataset), the air and soil temperatures showed the largest correlation coefficient (0.56); this correlation was much higher for some individual plots, with correlation coefficients of 0.72, 0.70, 0.56 and 0.46 respectively for plots GN, GL, CA and AL. On each individual year (considering data aggregated over all plots), the correlation coefficients were lower than 0.5, except in 2019 when the correlation coefficient was 0.79. In many cases, significant correlations were observed also between solar irradiance and both soil and air temperatures, thus air temperature and solar irradiance were excluded from the possible candidate predictors of *ER* when including soil temperature, and air and soil temperatures were excluded from the possible candidate predictors of *GPP* when including solar irradiance. Significant anti-correlations were also found between *DOY* and both soil moisture and soil temperature for most sites. The correlation between *DOY* and soil data points to the presence of a seasonal trend in environmental variables. For such reasons, the *DOY* was considered always as a last entry in the model, after having tested all the measured variables, in order to avoid using *DOY* as a proxy of a measured environmental variable. Concerning the hour of sampling, only sparse correlations were found with the solar irradiance, when considering either each year or each site. The general trend shown by correlations is confirmed by the analysis of partial correlations. This second type of correlations was calculated considering the variables in pairs and disregarding the influence of the remaining ones.

Different sets of uncorrelated additional predictors were tested for the measurements obtained in each individual year or in each individual plot, and for the whole dataset (all years and all plots). In nearly all cases, Eqs (1) and (2) were found to be the best descriptors. Only for *ER* in plot GN, the model using air humidity was marginally better than that based on soil moisture. However, the improvement in model performance was quite small, Δ*AIC* = 4.13, a value that is nearly negligible according to [28]. Hence, we always used Eqs (1) and (2) for the homogeneity of the comparison.

**Fig 2:**
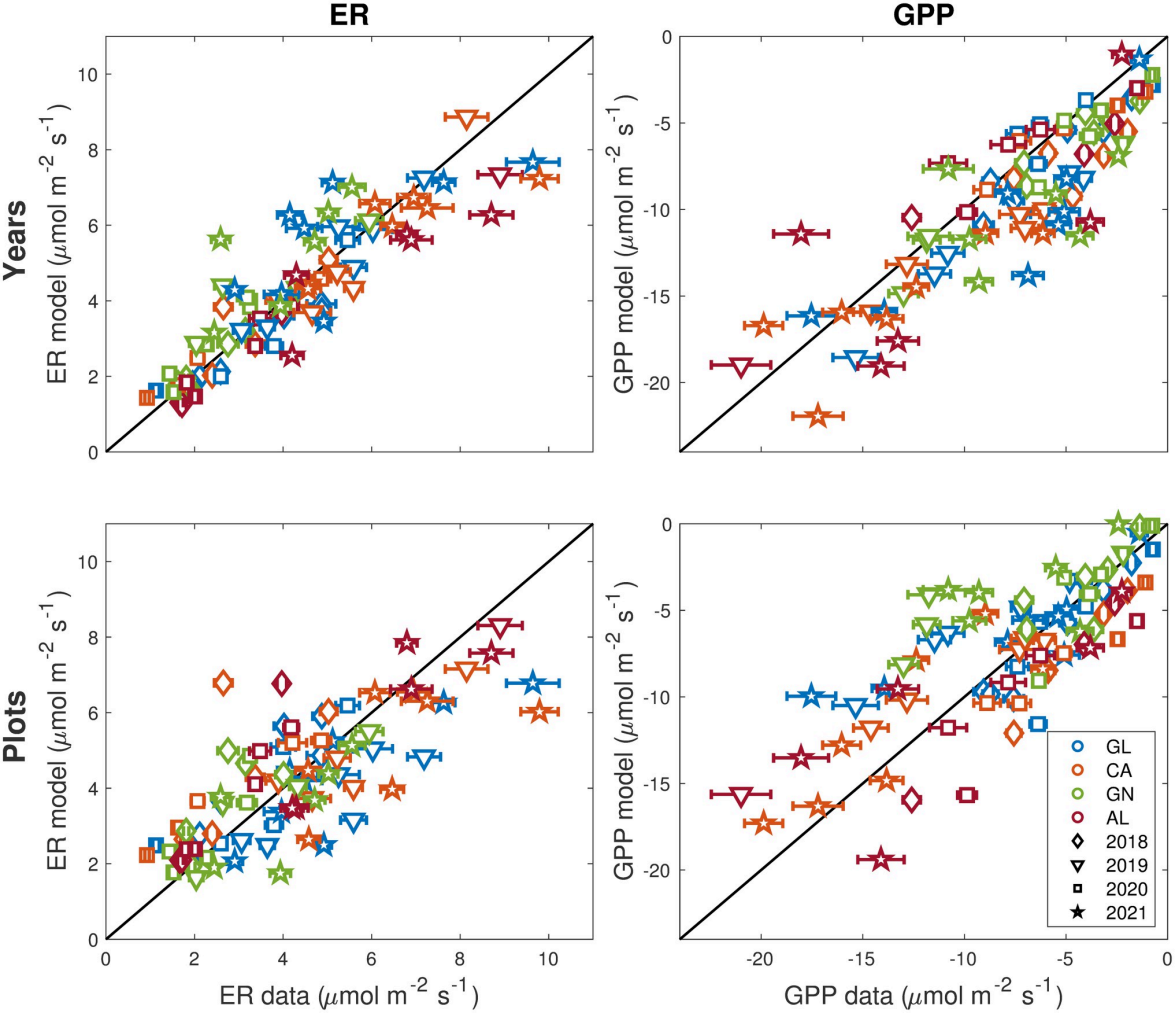
Modelled versus measured ER and GPP values estimated respectively using the models developed for each year (upper panels) and for each plot (lower panels). Diamonds are for the year 2018, triangles for 2019, squares for 2020 and stars for 2021. Colours correspond to plots GL (blue), CA (orange), GN (green), AL (red). The horizontal bars indicate the standard error on the mean (i.e. the standard deviation of the M = 20 point measurements divided by the square root of sample size, $\sqrt{M}$).

The *ER* and *GPP* model parameter values are reported in Table 2. For each year and each plot, the fit was significant (P<0.05 using the shuffling method) and the model residuals were gaussian according to Lilliefors’ test. In general, models for individual plots showed lower explained variances compared to models for individual years, in keeping with a larger year-to-year variability than plot-to-plot variations. This is also evident in Fig 2, where values in the top panels (model parameters estimated for each individual year) are closer to the diagonal than in the bottom panels (model parameters estimated for each individual plot). In Fig 2, we estimated the model parameters on the whole set of plots (when considering individual years) or years (when considering individual plots), but in the model-data comparison we kept track (using different colours) of which plot or year we are comparing. As mentioned in the Methods section, we could not fit the models to individual years and plots owing to the limited number of data points, and we were forced to aggregate either over plots or over years.

**Table 2:** Parameter estimates and explained variance (${\sigma^{2}}_{expl}$) for the models described by Eqs (1) and (2), respectively for ER and GPP.

		ER model—Eq (1)						GPP model—Eq (2)					
		b_0_	a_0_	a_1_	a_2_	a_3_	${\sigma^{2}}_{expl}$	F_0_	*α*_0_	A_0_	A_1_	A_2_	${\sigma^{2}}_{expl}$
year	2018	**0.12** *	**10.6**	-0.004	**-0.01**	**-0.006**	0.87	**-21.7**	-0.008	3.9	0.001	-0.01	0.68
	2019	**0.05**	9.6	**0.02**	-0.005	**-0.02**	0.85	**-8.7**	-1.5*^,^^+^	**3.2**	-0.007	**-0.01**	0.65
	2020	**0.04**	16.7	**0.08**	-0.015	**-0.02**	0.80	**-20.8**	-0.04	3.2	0.01	**-0.01**	0.88
	2021	0.03	**46.7**	-0.006	-0.05	**-0.03**	0.49	**-27.2**	-0.02	**4.2**	-0.006	**-0.01**	0.70
plot	GL	**0.06**	22.1	**0.04**	-0.02	-0.01	0.58	**-19.2**	-0.03	**1.7**	0.01	-0.006	0.54
	CA	0.03	4.2	0.06	0.004	-0.02	0.45	**-11.4**	-0.13	2.5	0.02*	-0.009	0.56
	GN	0.04	58.2*	0.01	-0.07*	**-0.02**	0.52	**-3.8**	-0.02	**9.0**	-0.03	**-0.03**	0.38
	AL	**0.05**	52.9	**0.11** *	-0.06	**-0.02**	0.87	**-5.8**	-0.05	**8.7**	0.02	**-0.03**	0.64
All		**0.04**	**29.4**	**0.03**	**-0.03**	**-0.02**	0.47	**-12.4**	**-0.02**	**3.9**	0.006	**-0.01**	0.46

Parameters are obtained either for each individual year (aggregating over all plots) or for each individual plot (aggregating over all years). The line ‘All’ indicates the values obtained fitting the model to the set of all plots (GL = Glacial; CA = Carbonate; GN = Gneiss; AL = Alluvial) and all years. Parameters that are significantly different from zero (P<0.05) are highlighted in bold. Units: °C^-1^ for b_0_; μmol m^-2^ s^-1^ for a_0_, a_1_, and F_0_; μmol m^-2^ s^-1^ hPa^-1^ for a_2_; μmol m^-2^ s^-1^ day^-1^ for a_3_; μmol W^-1^ s^-1^ for *α*_0_; d^-1^ for A_1_.

* parameter values that are significantly different from the value of the corresponding parameter estimated from the ensemble of the other remaining 3 plots or years

^+^ indicates parameter values that are significantly different from the value of the corresponding parameter estimated over all plots and years (line ‘All’)

To further explore the performance of the models, we estimated the root mean square error (RMSE) of the modelled fluxes with respect to the measured ones, using the parameter values reported in Table 2. Overall, the *ER* model developed either for each plot or for each year on average shows lower RMSE compared to the *GPP* counterpart, in agreement with the results of the explained variance.

### 3.3 Parameter differences

First, we compared pairs of plots or years (S3 and S4 Tables). In the comparison between pairs of plots, the only significant difference was observed in the dependence of *ER* on pressure, between plots GL and GN. On the other hand, the analysis revealed a marked variability of the parameters when comparing individual years, especially for *GPP*. In particular:

- between 2019 and the other years in the irradiance response of *GPP* (parameter *α*_0_);
- between 2020 and the remaining years, and between 2018 and 2019 in the light saturation level of *GPP* (parameter *F*_*0*_);
- between 2018 and the remaining years, and between 2019 and 2020 in the temperature response of *ER* (parameter *b*_*0*_)
- between 2018 and both 2019 and 2020 in the soil moisture response of *ER* (parameter *a*_*1*_).

This confirmed the larger variability between years (aggregating over plots) than between plots (aggregating over years) already observed in the mean values (Table 1). The difference in the estimated parameter values also suggests that the year-to-year variability was mostly related to changes in the response of the ecosystem fluxes to similar values of the drivers.

Since the estimated values of each parameter varied over a wide range, they could have significant differences between them but belong to the same distribution (e.g. values located in the low and high tail of the same distribution). Hence, we tested whether the model parameter estimates for one plot (or year) could be considered as a sub-sampling of the same parameter distribution, i.e. the distribution of parameters within the Nivolet plain, represented by the ensemble of all plots (or years). This was assessed by comparing the parameter values at a given plot (or year) with the parameter values obtained either at the remaining plots (years) or for the ensemble of all plots (years). Very sparse differences were obtained in this case, as indicated by the asterisks and crosses in Table 2, probably owing to the relatively large parameter distributions obtained when merging all data together.

## 4. Discussion

In this study we analysed the year-to-year and plot-to-plot CO_2_ flux variability measured in the Alpine high-altitude grasslands of the Nivolet Plain in the Gran Paradiso National Park, western Italian Alps. The mean ER values measured in this Alpine grassland across the four years (2018–2021) and in the four sampling plots were in the range of other studies located in Alpine sites, such as high-altitude Tibetan Alpine meadows (5.49 *μ*mol m^-2^ s^-1^ [29]), Swiss Alpine grasslands (5.2–6.5 *μ*mol m^-2^ s^-1^; [30]) and Colorado Rocky Mountains tundra (2.01 *μ*mol m^-2^ s^-1^; [31]) Similarly, GPP mean values agree with those of the Alpine grasslands on the Qinghai–Tibetan plateau where GPP daily fluxes vary between −8.78 *μ*mol m^-2^ s^-1^ and −5.4 *μ*mol m^-2^ s^-1^ [32]. Mean values of NEE are only slightly lower than those observed on the Qinghai–Tibetan plateau (−7.4 ± 0.9 and −6.7 ± 0.6 *μ*mol m^-2^ s^-1^ [32]) (see Table 1). In analogy with [33], the interannual variability of mean ER and GPP did not follow the annual pattern of their expected drivers: air/soil temperature and solar irradiance, respectively. Therefore, we analysed the flux drivers at a finer scale, using individual measurement campaign for each individual plot (aggregated over years), or for each year (aggregated over plots).

We adopted an empirical modelling approach that describes the dependence of the fluxes on different environmental drivers. Beyond the classical drivers (solar irradiance for *GPP* and soil temperature for *ER*), we confirmed the relevance of the additional flux predictors already identified in a previous study [21], namely [*VWC*, *DOY*] for *GPP* and [*VWC*, *Pr*, *DOY*] for *ER*. This set of drivers agrees with that used in other sites located in the European Alps [30, 34]. Similar predictors were also obtained for *ER* in a dry Alpine tundra in Colorado Rocky Mountains [31] and soil moisture played a relevant role also in a flux study in the Tibetan Alpine meadow [29, 35, 36]. Such correspondence suggests that similar drivers may be active in different geographical areas characterised by the presence of Alpine tundra and high-altitude grasslands. On top of climatic drivers, different indexes have been used to describe plant physiological processes during the growing season, thus supporting the interpretation of the *DOY* as a proxy for plant phenology. In a close site, [37] successfully integrated canopy greenness descriptors in a GPP model to describe plant phenology. In alpine grassland ecosystems in the European Alps [38] and Qinghai–Tibetan Plateau [39, 40], vegetation indexes had strong seasonal patterns, which may explain the role of DOY in our study. Because measurements mostly started when the soil was completely free from snow and the vegetation was already green, only the declining trend toward senescence of vegetation was observed, which is also signalled by the negative values of the parameters *a*_*3*_ and *A*_*2*_ for ER and GPP model (Table 2), respectively.

At the landscape scale, the interannual variability of CO_2_ emission and uptake in the period 2018–2021 was stronger than its spatial variability, associated with the selection of plots characterised by soils of different origins within the same watershed (plots GL, CA, GN, AL). The models developed for each individual year showed higher explained variances compared to the models developed for each plot (Table 2). In addition, the difference of model parameters was stronger and more significant between the different years than the different plots. These results were in contrast with our first hypothesis (i.e., significantly different flux behaviour between plots), but confirmed the second hypothesis (i.e., significantly different flux behaviour between years). Thus, the same parameter values captured the specific characteristics of the different plots better than the flux variability across different years (Fig 2). This suggests that climatic and phenological factors are the main controls on primary production and ecosystem respiration, while in our study case the plot-dependent factors (e.g., soil properties and underlying rock type) seem to play a minor role.

Similar results were obtained for CO_2_ emissions measured in different sites located in the Swiss Alps [34]. These authors compared the temperature response of *ER* across different soil and vegetation types, observing non-significant differences except for young fluvisols and *Petasition* species. Consistently, the alluvial plot (AL) in our study showed the highest RMSE in the models estimated for each individual year. In addition, the same study [34] also highlighted a stronger influence of soil type on heterotrophic rather than on autotrophic respiration, suggesting that the latter could be dominant at our study site. The numerous similarities with independent studies performed in other locations of the European Alps support the generality of the results discussed here and suggest that an empirical model such as the one developed here could be upscaled to broader scales for high-altitude Alpine tundra and grassland environments.

Although interannual variability of CO_2_ fluxes has already been observed in other works, this has not yet been attributed univocally to a specific mechanism. Such variability was linked to annual precipitation patterns or variations in solar radiation [41], to temperature and precipitation [42] while in some work it was unclear what were the main contributing factors [43]. In our study, the strong interannual variability was not merely associated with variations of the drivers across years (Table 1). Indeed, significant differences were observed between the model parameters estimated for the different years (S3 and S4 Tables). In other words, provided the same structure of the equation, which represents the underlying biophysical system (i.e., the vegetation and the soil microbiota) behaviour, and the same value for the drivers, different parameter values result in different flux values. Hence, the observed change in the model parameters expresses a different response of the system to the drivers, which is not trivially justified by interannual changes in the drivers themselves, in agreement with [33, 44].

The significant difference of model parameter values across years may be ascribed either to descriptors that are not accounted for in the model, or to the fact that measured fluxes may depend on the specific history of the system and therefore on past values of the drivers. Regarding the first possibility, biomass, leaf area index, composition of vegetation communities and soil carbon [31, 45–47], which can vary from year to year depending on the specific response of the biota to the climatic conditions, could influence the parameter values of the empirical models discussed here, and will be object of future measurements. Regarding the second possibility, plant desiccation for drought stress, precipitation seasonality or past plant activity [31, 45, 48], as well as microbial community shifts (e.g. from bacterial dominated to fungal dominated communities, or vice versa) in response of high temperature and droughts [3, 4] could change the dynamics of the system, thus modifying the subsequent efficiency of carbon exchanges with respect to other years [49].

## 5. Conclusions

In this work we analysed the spatial and interannual variability of carbon dioxide fluxes in the Alpine high-altitude grasslands in the Gran Paradiso National Park, western Italian Alps, considering a set of four plots, characterised by different underlying rock types in the same watershed, where measurements were carried out over four years. We first explored the mean values of meteo-climatic and environmental variables and then identified which of them acted as driving factors of the CO_2_ fluxes at landscape scale. A main result of the analysis is that in the high-altitude alpine grasslands considered here, the interannual variability of CO_2_ fluxes is predominant over the plot-to-plot variability. Our results suggest that in this environment the CO_2_ flux dynamics is more sensitive to the interannual variability of climatic conditions than to plot-specific characteristics. Therefore, using similar parameterizations across different sites may allow for the upscaling of carbon exchanges and produce reliable estimates of carbon fluxes at landscape scale. On the other hand, long-term monitoring emerges as a necessary requirement to characterise the significant interannual variability of carbon flux dynamics in this environment.

## Supporting information

S1 Fig Soil properties in the first horizon (type A, 0–10 cm depth) across the four plots. Soil samplings were performed in July 2018. Soil texture was reported in panel A; while soil organic carbon (SOC) and total nitrogen (TN) were reported in panel B.
(PDF)

S1 Table Variables showing significant differences between years 2018, 2019, 2020, 2021, aggregating over all plots. (PDF)

S2 Table Variables showing significant differences between plots GL, CA, GN, AL, aggregating over all years. (PDF)

S3 Table Parameters showing significant differences (P<0.05) between different years for *ER* and *GPP*, aggregating over all plots. (PDF)

S4 Table Parameters showing significant differences between plots for *ER* and *GPP*, aggregating over all years. The only marginally significant difference is observed for *ER* between GN and GL, with a P-value P = 0.05.
(PDF)
