# Supplementary material for: Spatial and temporal variability of carbon dioxide fluxes in the Alpine Critical Zone: The case of the Nivolet Plain, Gran Paradiso National Park, Italy
Source: PLoS One. 2023 May 30;18(5):e0286268. doi: 10.1371/journal.pone.0286268 (PMC10228792; doi:10.1371/journal.pone.0286268)
Supplement: S1 Fig — Soil samplings were performed in July 2018. Soil texture was reported in panel A; while soil organic carbon (SOC) and total nitrogen (TN) were reported in panel B. (PDF) [file pone.0286268.s001.pdf]

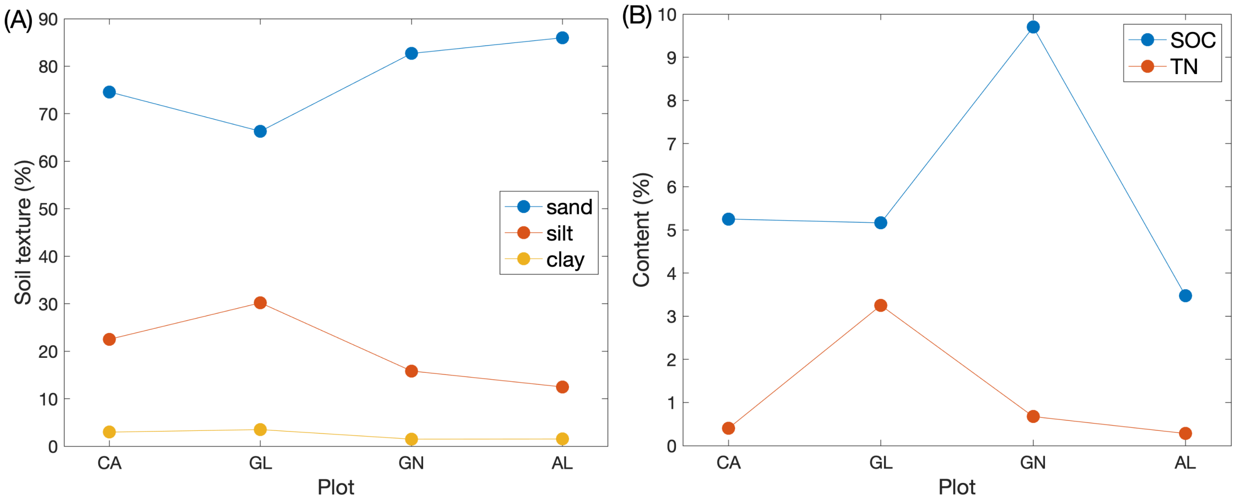

Figure S1. Soil properties in the first horizon (type A, 0-10 cm depth) across the four plots. Soil samplings were performed in July 2018. Soil texture was reported in panel A; while soil organic carbon (SOC) and total nitrogen (TN) were reported in panel B.
