# Supplementary material for: Spatial and temporal variability of carbon dioxide fluxes in the Alpine Critical Zone: The case of the Nivolet Plain, Gran Paradiso National Park, Italy
Source: PLoS One. 2023 May 30;18(5):e0286268. doi: 10.1371/journal.pone.0286268 (PMC10228792; doi:10.1371/journal.pone.0286268)
Supplement: S1 Table — (PDF) [file pone.0286268.s002.pdf]

Table S1. Variables showing significant differences between years 2018, 2019, 2020, 2021, aggregating over all plots.

	2019	2020	2021
**2018**	T_s, q	VWC, NEE, ER, GPP	T_s, T_a, VWC, rs, ER, GPP
**2019**	-	T_s, T_a, rs, NEE, ER, GPP	VWC, Pr, ER, GPP
**2020**	-	-	T_s, T_a, VWC, rs
