# Supplementary material for: Spatial and temporal variability of carbon dioxide fluxes in the Alpine Critical Zone: The case of the Nivolet Plain, Gran Paradiso National Park, Italy
Source: PLoS One. 2023 May 30;18(5):e0286268. doi: 10.1371/journal.pone.0286268 (PMC10228792; doi:10.1371/journal.pone.0286268)
Supplement: S2 Table — (PDF) [file pone.0286268.s003.pdf]

Table S2. Variables showing significant differences between plots GL, CA, GN, AL, aggregating over all years.

	CA	GN	AL
**GL**	VWC, Pr	rs, Pr, ER	VWC, Pr
**CA**	-	Ts, rs, Pr, ER	Ts, rs, Pr
**GN**	-	-	Pr, GPP
