# Supplementary material for: Spatial and temporal variability of carbon dioxide fluxes in the Alpine Critical Zone: The case of the Nivolet Plain, Gran Paradiso National Park, Italy
Source: PLoS One. 2023 May 30;18(5):e0286268. doi: 10.1371/journal.pone.0286268 (PMC10228792; doi:10.1371/journal.pone.0286268)
Supplement: S3 Table — (PDF) [file pone.0286268.s004.pdf]

Table S3. Parameters showing significant differences (P<0.05) between different years for *ER* and *GPP*, aggregating over all plots.

	ER			GPP		
	2019	2020	2021	2019	2020	2021
2018	b_0, a_1	b_0, a_1	b_0	F_0, α_0	F_0	-
2019		b_0	-		F_0, α_0	α_0
2020			-			F_0
