# Supplementary material for: Spatial and temporal variability of carbon dioxide fluxes in the Alpine Critical Zone: The case of the Nivolet Plain, Gran Paradiso National Park, Italy
Source: PLoS One. 2023 May 30;18(5):e0286268. doi: 10.1371/journal.pone.0286268 (PMC10228792; doi:10.1371/journal.pone.0286268)
Supplement: S4 Table — The only marginally significant difference is observed for ER between GN and GL, with a P-value P = 0.05. (PDF) [file pone.0286268.s005.pdf]

Table S4. Parameters showing significant differences between plots for *ER* and *GPP*, aggregating over all years. The only marginally significant difference is observed for *ER* between GN and GL, with a P-value P=0.05.

	ER			GPP		
	GL	GN	AL	GL	GN	AL
CA	-	-	-	-	-	-
GL		a_0, a_2	-		-	-
GN			-			-
